# Orientation determination by wavelets matching for 3D reconstruction of very noisy electron microscopic virus images

**DOI:** 10.1186/1472-6807-5-5

**Published:** 2005-03-02

**Authors:** Ali Samir Saad

**Affiliations:** 1 Department of Biomedical Technology, College of Applied Medical Sciences, King Saud University, P.O. Box. 10219, Riyadh 11433, Kingdom of Saudi Arabia

## Abstract

**Background:**

In order to perform a 3D reconstruction of electron microscopic images of viruses, it is necessary to determine the orientation (Euler angels) of the 2D projections of the virus. The projections containing high resolution information are usually very noisy. This paper proposes a new method, based on weighted-projection matching in wavelet space for virus orientation determination. In order to speed the retrieval of the best match between projections from a model and real virus particle, a hierarchical correlation matching method is also proposed.

**Results:**

A data set of 600 HSV-1 capsid particle images in different orientations was used to test the proposed method. An initial model of about 40 Å resolutions was used to generate projections of an HSV-1 capsid. Results show that a significant improvement, in terms of accuracy and speed, is obtained for the initial orientation estimates of noisy herpes virus images. For the bacteriophage (P22), the proposed method gave the correct reconstruction compared to the model, while the classical method failed to resolve the correct orientations of the smooth spherical P22 viruses.

**Conclusion:**

This paper introduces a new method for orientation determination of low contrast images and highly noisy virus particles. This method is based on weighted projection matching in wavelet space, which increases the accuracy of the orientations. A hierarchical implementation of this method increases the speed of orientation determination. The estimated number of particles needed for a higher resolution reconstruction increased exponentially. For a 6 Å resolution reconstruction of the HSV virus, 50,000 particles are necessary. The results show that the proposed method reduces the amount of data needed in a reconstruction by at least 50 %. This may result in savings 2 to 3 man-years invested in acquiring images from the microscope and data processing. Furthermore, the proposed method is able to determine orientations for some difficult particles like P22 with accuracy and consistency. Recently a low PH sindbis capsid was determined with the proposed method, where other methods based on the common line fail.

## Background

Three-dimensional (3D) reconstruction of virus particles like SARS (Severe Acute Respiratory Syndrome) and HSV (Herpes Simplex Virus) using electron microscopy yields crucial information for understanding the assembly and infectivity mechanism. The structural determination begins with acquisition of projection images in an electron-microscope. A major part of data processing is aimed at determining the direction of projection for each particle image (2D projection of virus) so that a 3D reconstruction can be computed. The first step in a virus reconstruction is the detection and selection of the individual particle images from a large area of an electron micrograph.

**Figure 1:**
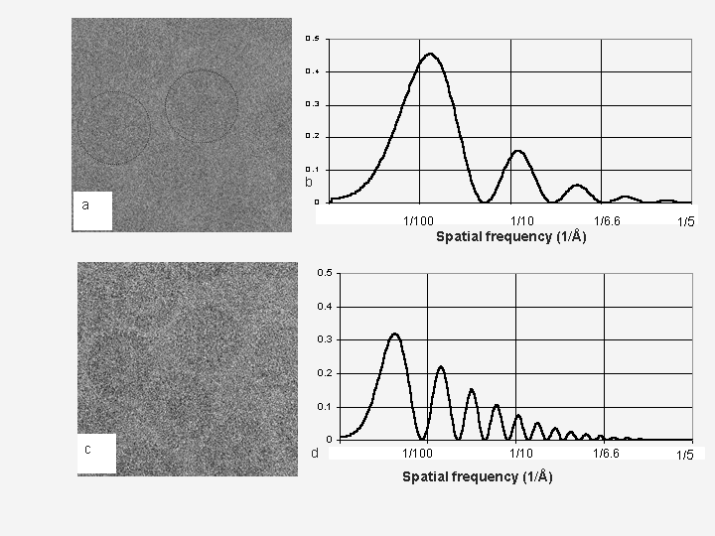
**Different defocus images with their spatial frequency resolution. **Figure 1 -a shows a typical image taken at 0.8 μm defocus with JEOL 400 kV electron cryo-microscope with a LaB6 gun. This image has low contrast but contains a detectable signal above noise at a relatively high spatial frequency out to 1/7 Å^-1 ^(b). The image contrast can be enhanced by setting the defocus value to a larger value (i.e. 2.6 μm as shown in c). However, such an image contains much lower resolution data as shown in (d), the detectable signal is present out to spatial frequency (1/12Å^-1^). In order to obtain a high-resolution reconstruction, we would use images taken at condition similar to that of (a).

There are different criteria to determine the particle orientation. One criterion is based on the computational search of the common lines in the computed Fourier Transforms of individual or multiple particle images [1]. An improvement of the Fourier Common Line algorithm [2] has been proposed, but a significant amount of the low contrast particle images are still discarded, partly because of the impossibility of obtaining a reliable estimate of their orientations. Another criterion for the particle orientation estimate is to find the correlation match between the raw images with many projections from a 3D model [3]. Regardless of the criterion used, finding the orientation determination for a particle image such as that in Fig. 1a is difficult because of its extremely low contrast.

One approach is to take two consecutive pictures of the same particles one close-to-focus (Fig. 1-a) and another farther from focus with a higher contrast (Fig. 1-c) from which the initial orientations are easily determined [4,5]. The initial orientations are then assigned to the corresponding particles in the close-to-focus images for structural refinement (henceforth, called focal pair method). In a high resolution structure determination, one would require over 6000 particles of data for 8.5 Å resolutions [6]. If a focal pair is required, one would need over 12,000 particles and hence it is a labor-intensive process of data recording, digitization and archiving. In this paper, we propose a method for determining the initial orientations of the particles from low contrast (close-to-focus) images without necessity for a second set of highly defocused images. In this technique, we use the wavelet transformation in a multi-resolution analysis [7,8] to enhance the contrast of the image and the hierarchical weighted projection matching to accelerate the processing. The wavelet-transformed images have the same size as the original images. Wavelet decomposition separates the low-resolution information, called "approximation", from the high resolution information, called "details". This method computationally generates an image equivalent to the far-from-focus picture taken by the microscope and separates images containing details and noise. The technique proposed here is a model-based approach in wavelet space, which we call Hierarchical Wavelet Projection Matching (HWPM).

## Results

A data set of 600 HSV-1 capsid particle images in different orientations was used to test the HWPM method. The defocus range of herpes particles was chosen to be close to focus between 1.7 μm and 0.4 μm. An initial model of about a 40 Å resolution [4] was used to generate projections uniformly covering the asymmetric triangle of the icosahedrally symmetric HSV-1 capsid particle [4,5]. A grid sampling of 0.5° in each direction of the asymmetric triangle of icosahedral particles was used. The number of projections obtained with this grid was relatively high (2616 projections). First, the 2616 projections were grouped into 200 classes, each class containing about 13 projections.

A match of the particle into the best 3 of the 200 classes was obtained using the wavelet correlation coefficient (wccf) criterion. Next, the particle was compared to the 39 projections of the best three classes, and the correct orientation was that of the projection giving the highest wccf. The hierarchical implementation wavelet projection matching reduced the time at least by a factor of 10 compared with the classical projection matching method. In the example of 600 particles, by using HWPM it took approximately 3 hours to determine the orientations, instead the 33 hours it took with the classical matching algorithm. Both algorithms were running on the SGI Origin-2000 supercomputer using 10 processors.

At this point, each particle had been assigned the orientation of the closest projection. A quality factor was assigned to each orientation, which was the wavelet correlation coefficient. Particles having high wccf coefficients were selected for reconstruction of a first 3D model of the virus.

Refinement of initial orientations obtained by HWPM was realized by the same iterative refinement process used in focal pair method [5]. This refinement process uses both local and global refinement. Local-refinement refines orientations against a set of projections from the 3D density map. In global refinement, all the raw particle orientations are refined against each other, without using projections from the 3D model. A potential merit of global refinement is the absence of possible bias arising from the 3D model.

In order to assess the accuracy of the orientations obtained with the HWPM. A comparison with the focal pair method (Fig. 5.a), which is currently the most appropriate method for low contrast virus images was accomplished. The following steps were executed. First the initial orientations of the far-from focus particles were determined by using the cross-common line method between real particles and a set of projections obtained from the low resolution model. Next, a global refinement process was realized in order to determine the initial orientation. The same software as in [4] was used with the same initial parameters. The parameters used in this software were the minimum radius and maximum radius limiting the resolution and the sampling step size of 4.67Å/pixel. The minimum valid radius ensured that the minimal radius was computationally accurate when the two common lines angles were close and also to avoid the biasing of the orientations of particles by the very low frequency components. This parameter choice for herpes at the above sampling step was 5 pixels. The maximum radius was chosen to limit the maximum resolution expected from the reconstruction, here in the initial orientation the maximum radius corresponding to a resolution about 40 Å. Then an assignment of the particle orientations from the far to focus to the close to focus is realized. Next, an iterative refinement process to the close-to-focus data was accomplished as described in [5]. A 3D reconstruction using the best 300 HSV-1 particle orientations was performed for each method. Fig. 5.a and Fig. 5.b show surfaces density contour, displayed at one standard deviation above the mean density [9], obtained respectively from focal pair method and HWPM method. Both structures show a similar visual resemblance. In order to assess the reliability of the 3D density maps and the quality of particles orientations obtained from each method, the Fourier Shell Correlation (FSC) criterion, which is the most robust criterion [10,11], was employed. The FSC was calculated between 2 independent reconstructions from the same set of orientations for each method. The effective resolution assessment of the 3D structure obtained from each method is estimated at FSC correlation value of 0.5, which correspond to 45° phase difference.

Fig. 6 shows three different plots. The green curve shows a resolution of 32 Å of the reconstruction using the best 300 particles with orientations obtained from the focal pair method. The blue curve shows a resolution of 24 Å of the reconstruction using 300 particles with orientations obtained from the HWPM method. This result shows that the resolution of the structure obtained from the HWPM is higher than the one using the orientations from the focal pair method. Therefore, the orientations obtained from HWPM method are more accurate. Furthermore, HWPM method uses only one set of close-to-focus data instead of the two sets used by the focal pair method. The purple curve shows a resolution of about 14.5 Å of the reconstruction using 500 particles with orientations assigned by HWPM. The red curve plots twice the expected FSC for Gaussian noise. A less stringent criterion to assess the resolution as the intersection between the FSC curve and the curve plotting the 2 times expected Gaussian noise.

HWPM was tested on a P22 empty shell capsid which was circular and whose shell is very thin (~40 Angstrom). Twenty micrographs of the P22 empty shell capsid with defocus range [0.5 to 2 μm] were used for testing purpose. The total number of particles is 1340, each image has a size of 300 × 300 pixels, and the dimension of each pixel is 2.8 Å.

Concerning the initial orientations determinations using HWPM method, an initial model of around 20 Å resolutions was used to generate projections which uniformly covered the asymmetric triangle of the icosahedrally symmetric model. A grid sampling of 2° in each direction of the asymmetric triangle of icosahedral symmetry was used to obtain an initial orientation, targeting a structure of 30 Å. The number of projections obtained with this grid was about 200 projections. A match of the particle with the projections was obtained by using the wccf criterion. The correct orientation was selected as the one of the projection giving the highest wccf. The better half of the orientations projections (650) according to wccf criterion was chosen for final reconstruction.

The initial orientations for the same set of data were determined using the Improved Common Line (ICL) method, with the same input parameters for the software described in [2]. ICL use one single micrograph and does not use focal pair technique. The best half of the particles orientations (650) was chosen, according to the phase residual criterion, in the 3D reconstruction of the P22.

Fig. 7 shows three surface views of the P22 empty shell capsid. The Top image shows the original surface [12,13]. The lower right image shows the surface obtained by HWPM, which shows a very similar view to the original structure. The resolution assessment of the structure, by Fourier shell correlation criterion, gives a resolution of 14.5 Å. The lower left surface shows the result obtained by ICL method. The surface view of the reconstruction obtained from the ICL of the P22 empty shell capsid is different from the original P22 capsid. Fig. 7 proves the inaccuracy of some of the initial orientations obtained from the ICL method for such a smooth virus.

## Discussion

During the last thirty years the common lines methods were a great method to resolve icoshedral particles up to 7–8 Å [6]. Recently, a method using polar transformation and projection matching were used for the purpose of orientation determination [3], but this last method is not suitable for the high resolution of large virus because the resulting transformed images, could be double the size of the original image. The proposed method combines the projection matching of wavelet denoising for an initial determination of particle orientation, with the common lines method for refinement to a higher resolution. It is clear that HWPM method works only if the initial low resolution model of the particle is already known. This method is very interesting if we need to add more particles to an existing intermediate resolution reconstruction in order to increase the resolution. Particles having high resolution information are very noisy [9,14]. The best that we can get using the ICL method is less than 40 % of good orientations, for defocus values between 1.9 μm and 1.2 μm, for the P22 capsid [2]. Usually, very high resolutions use defocus values which go much lower than 1.2 μm as in the HSV data, or the current P22 data which goes to 0.5 μm. The 40% rate of correct orientations would certainly become smaller if we used data at closer defocus. The study accomplished on high resolution for HSV reconstruction showed that using a close-to-focus single micrograph with CL method was not effective, because a small number of orientations were found to be correct [9], for this reason a focal pair method was used for 8.5 Å structure[9].

At high resolution reconstructions, the number of particles needed increases drastically, and the data with a signal-to-noise ratio valid up to the targeted resolution, tend to be very noisy. For an 8.5 Å structure of HSV-1 it took about 6000 particles for a final reconstruction. For a 6.5 Å structure resolution, the estimated value was about 50,000 particles using the same electron microscope [14].

To further increase the resolution of the HSV virus to 6.5 Å or higher (4 Å), the focal pair method would be impracticable. The focal pair method, for intermediate resolution up to (8 Å) for big viruses like HSV, works well for orientations determinations. The number of particles selected for the final reconstruction about 40% of the original number of particles (taking into account the far-focus and close-focus micrographs). It is necessary to emphasize that results from both methods are very similar in terms of visual resemblance. But, there are two advantages of HWPM over the focal pair method. First, focal pair method uses as much as double the data used for the HWPM. Second, the quality of the density maps shows that HWPM gives a better resolution for the same number of particles (figure 6). This proves a better accuracy of orientations determinations obtained by the HWPM.

One of the more obvious advantages of the HWPM for orientation accuracy appears in two examples of real reconstructions. The first is for the P22 capsid, the ICL method does not give a good initial orientation, and the refinement of the orientations does not help to converge toward the right orientations. The probable reasons why the ICL method did not work properly for the P22 capsid are: first the P22 capsid has a smooth surface (the thickness of the shell is about 40 Å); second most of the data are very close-to-focus with defocus range of 0.5 μm to 1.3 μm. The data was noisy and had a very low contrast. The ICL method was able to give 40% of good orientations for the defocus range between 1.9 and 1.2 μm, here the data was closer to focus, which reduced the percentage of good orientations to less then 22%.

The application of the HWPM to the P22 empty shell capsid gave the expected structure (Fig. 7). The wavelet denoising in the HWPM not only helped in reducing the noise and enhancing the contrast of the particles, but also used the entire information from the image (instead of using several lines) which is enhanced accuracy for highly noisy particles.

Another example of real data reconstruction is the VP5-VP19C recombinant. After long investigation using CL and ICL algorithms, the classical projection matching scheme was also tested in order to determine the orientations, but unfortunately all those methods failed. The wavelet filtering and matching was used during the classification step of the recombinant particle VP5-VP19C [15,16], which significantly improved the quality of the class averages [16-18] and enabled the determination of the structure of that particle. A study [16] shows the superiority of the wavelet projection matching over the Gaussian filtered projection matching.

The third examples for low PH sindbis: Three years of investigation using CL and ICL methods failed to obtain the correct density map of the low PH sindbis capsid which is subject to conformational changes and an alteration of the symmetry. Recently the proposed method (HWPM) was tested on low PH sindbis and the correct structure was finally observed and analyzed [19].

Wavelet multi-resolution analysis and processing improves particle detections [8], classification [15,16], and orientation determination on a variety of electron microscopy images which are highly noisy and have an extremely low contrast. This prove that wavelet techniques are adequate in the 3 main steps of 3D virus reconstruction and in the classification step of single particle reconstruction [16,17].

## Conclusion

This paper describes the development and implementation of a new method for orientation determination for low contrast images of virus particles. This method is based on wavelet filtering, which enhances the contrast of the particles and reduces the noise, and on weighted projection matching in wavelet space. A hierarchical implementation of this method increases the speed of orientation determination. Results show that, HWPM have been able to determine accurately more than 85% of the orientations of low-contrast particles. Compared to the focal pair method (for orientation determination from low contrast data) the HWPM reduced the amount of data required in a reconstruction by at least 50 %. In addition the accuracy of the orientations obtained by the proposed method is higher than those obtained by focal pair method [9]. This improved accuracy is shown clearly by the resolution assessment in Fig. 6. The estimated number of particles needed for a 6.5 Å reconstruction of the HSV-1 capsid was about 50,000 [14]. By using the HWPM method, only half as much data was necessary. The proposed method could save 2 to 3 man-years invested in acquiring images from the microscope and data processing. Another advantage of this method is the ability to give accurate orientations for some particles having conformational changes or alteration of symmetry as seen for VP5-VP19C recombinant and recently with the low PH sindbis capsid.

## Methods

### Choice of wavelet Base

The choice of wavelet filter bases depends on the signal. Signals coming from different sources have different characteristics. For audio, speech, image and video signals the best choices of wavelet bases are known. The best choice for electron microscopic images is not clear. The problem is to represent typical signals with a small number of convenient computable functions.

An investigation to choose the best wavelet bases for electron microscopic images was performed here. During this study, simulated and real electron microscopy images were used. The majority of the wavelets basis existing in Matlab-5 software [20-24] was tested. The criterion used to determine the best wavelet base was the one which optimizes the signal to noise ratio in a broad spectrum of spatial frequencies. The bi-orthogonal wavelets basis [25-27] especially the 3.5 basis in Matlab-5 yielded the best average signal to noise ratio in the range of the spatial frequency (1/100 - 1/8 Å^-1^) relevant to data analysis.

### Wavelet Projection Matching (WPM) Principle

**Figure 2:**
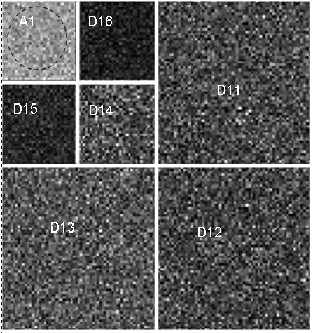
**Wavelet decomposition of a virus at level 2. **Level 2 decomposition of wavelet uses bi-orthogonal base. This transformation is applied on a projection of phi 13 model downloaded form the Proteins data bank and then reconstructed to 10 Å.

The principle of the wavelet decomposition is to transform the original raw particle image into several components: one low-resolution component called "approximation" [21], which is mainly used in this method, and the other components called "details" (Fig. 2).

The approximation component is obtained after applying a bi-orthogonal low-pass wavelet filter in each direction (horizontal and vertical) followed by a sub-sampling of each image by a factor of 2 for each dimension. The details are obtained with the application of a low-pass filter in one direction and a high-pass filter in the other, or a high-pass filter in both directions. The noise is mainly present in the detail components. A higher level of decomposition is obtained by repeating the same filtering operations on the approximation. The wavelet correlation coefficient between two wavelet-transformed images, for a given level, is :


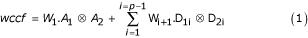


Where W_1 _to Wp are weights given for each components of the wavelet correlation, p is the number of components of wavelet decomposition. A_1_, A_2 _are the approximations. ⊗ denote the correlation between two components images. D_1i_, D_2i _are the details (Fig. 2). This implementation starts first by a wavelet filtering which is performed by thresholding [21,28,29] of the details components in order to reduce the noise effects in the correlation matching. Higher weight is given to the approximation component to further reduce the noise effect in the decision. The weights given in this implementation are 0.75 for the approximation and 0.25 for the details.

### Orientation determination with Hierarchical WPM (HWPM)

**Figure 3:**
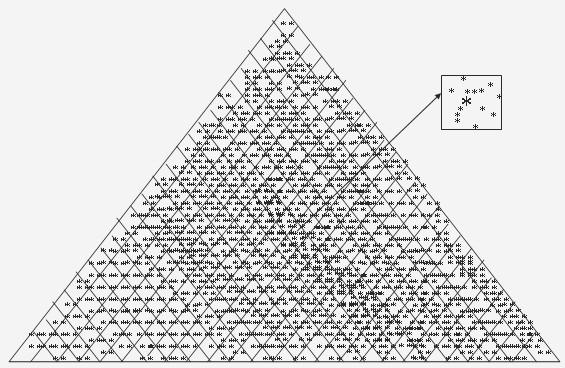
**Classification scheme of projections into classes. **Classification of projections using (2 of the 3) Euler angles for viruses having Icosahedral symmetry like the herpes virus. Only an asymmetric triangle is needed in the case of an Icosahedrally symmetric virus instead of the whole plan in the case of an asymmetric object. Each rectangle represents a class of projections. The closest projection to the center of the rectangle, represents the center of the class. This figure represents the classification of 2600 projections into 300 classes.

Initial orientation determination is based on model-based projection matching approach [3]. The level of wavelet decomposition depends on the dimension of the virus and the sampling rate. For herpes simplex virus type-1 (HSV-1) B-capsid, which has a diameter of 1250 Å with a sampling of 2.1 Å/pixel, a level two of wavelet decomposition (Fig. 2) is appropriate for the initial orientation estimate, because of the contrast enhancement and the consideration of computational speed. The method starts by generating the wavelet decomposition at level two for each projection and raw image. In order to have accurate orientation estimation a small angular grid (figure 3) to generate projections from the initial model is needed, and this results in a large number of projections. The classical projection matching, which consists of comparing the wavelet-transformed raw images with every projection, is very slow even when using multiple processors on a parallel computer. In order to significantly increase the speed of processing, a hierarchical implementation is performed. This consists of grouping projections into classes of similar orientations [30]. Fig. 3 shows the classification scheme applied for the icosahedral viruses, only an asymmetric triangle representing the possible orientations for icosahedrally symmetric object [4] is considered.

**Figure 4:**
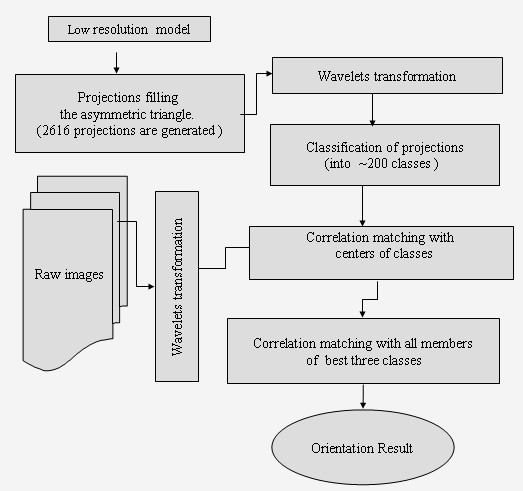
**Schematic diagram of the HWPM method. **Schematic diagram of the HWPM method: The first step is the wavelet decomposition of each of the particles, and projections and then classification of projections into 200 classes. The final step is to determine the correlation by using the centers of classes and then using all members of the best 3 classes to get the best orientation estimates.

**Figure 5:**
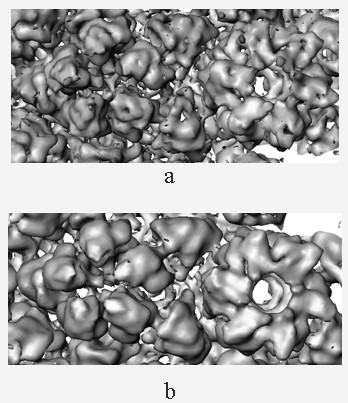
**Comparison between 3D reconstruction obtained from both methods. **Reconstruction of the B-capsid of the herpes simplex virus type-1: in (a) the initial orientations are determined from the second picture and then refined using the first picture. In (b) Initial orientation is determined from the wavelet-projection matching algorithm with one refinement iteration using the common line program. Both iso-surfaces are displayed at 1 standard deviation above the mean.

**Figure 6:**
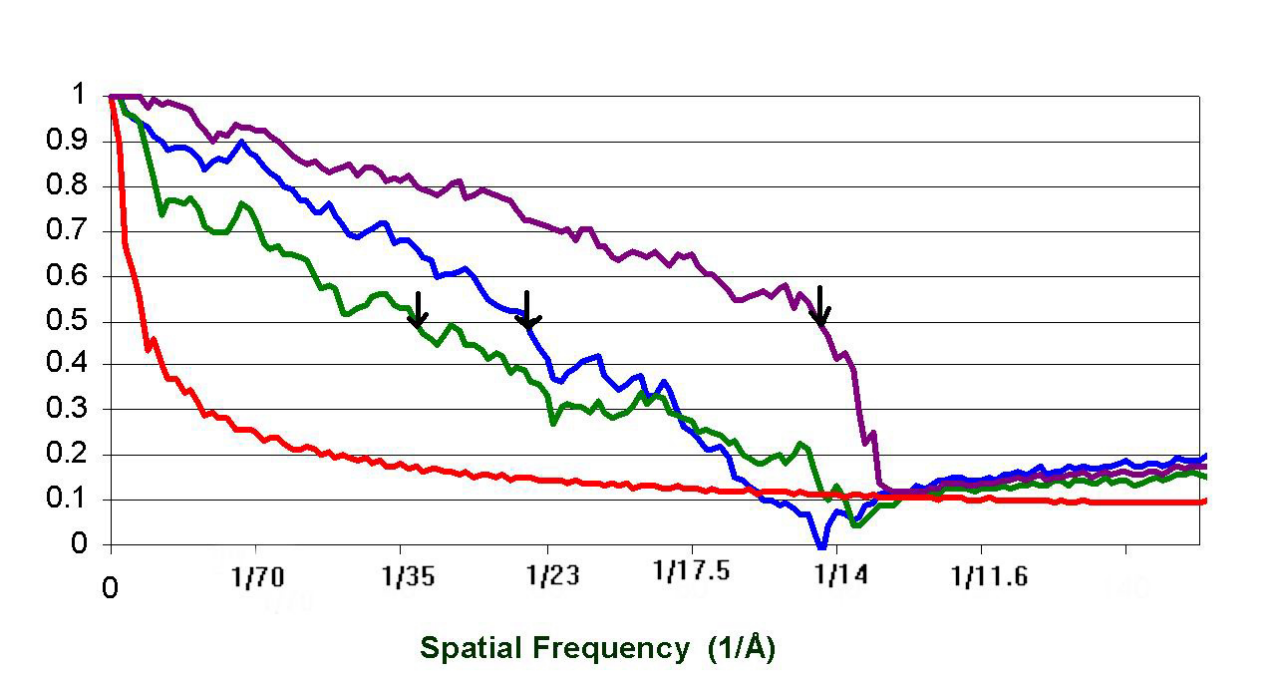
**FSC between 2 independent reconstruction for each method. **Green and blue lines show the correlation as function of the spatial frequency using 300 particles from the Focal pair method and HWPM respectively. Using the 0.5 correlation point criterion to assess the resolution of the 3D structure, the dotted line gives a resolution of 32 Å and thin line gives a resolution of 24 Å. The purple line shows a reconstruction using 500 particles where the orientation is obtained from the HWPM method the estimated resolution using 0.5 correlation point shows 14.5Å. The red line shows the 2 times expected FSC for Gaussian noise.

**Figure 7:**
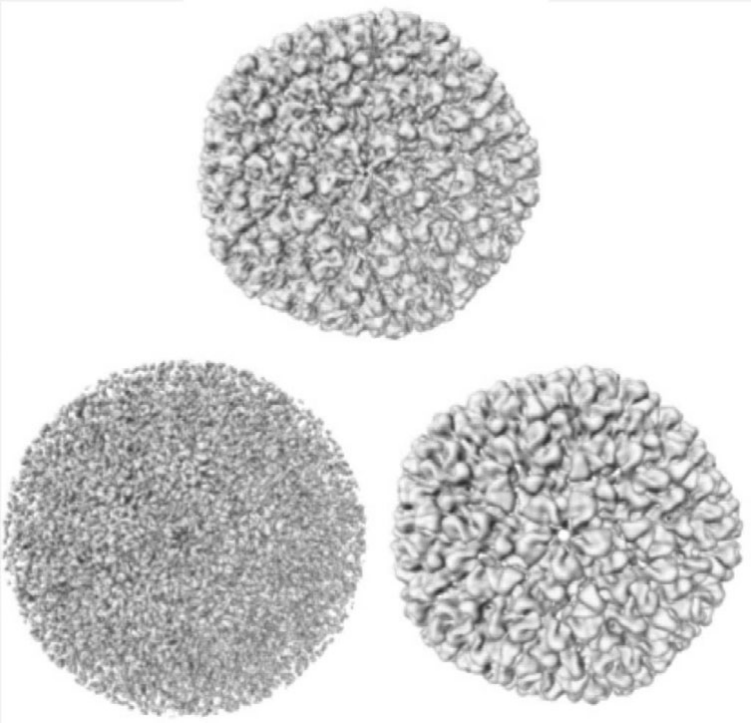
**Surface visualization of P22 empty shell capsid. **For surface visualization of P22 empty shell capsid, three views were taken at the 5 fold direction and surfaces were displayed at 1.5 standard deviations above the mean. Top image is the original surface from Zhang [13], the lower left image is the reconstruction obtained from ICL, the lower right image is the reconstruction obtained from the proposed method.

The choice of the number of classes is optimized to give the best tradeoff between speed and accuracy. The classification gives a uniform distribution of projections into the classes. The next step is to compare each wavelet-transformed raw image with the closest projection to the center of each class, and then rank the classes in terms of wccf (Fig. 4). The final step is to compare the raw image with all the projections of the three classes given the highest wccf coefficients. Next, the orientation of the projection yielding the highest wccf will be assigned to the raw image as the correct orientation. The software is written in C++ (a parallel version of the software has been written to run on the SGI Origin-2000 supercomputer).
